# Supplementary material for: An Angelman syndrome substitution in the HECT E3 ubiquitin ligase C-terminal Lobe of E6AP affects protein stability and activity
Source: PLoS One. 2020 Jul 8;15(7):e0235925. doi: 10.1371/journal.pone.0235925 (PMC7343168; doi:10.1371/journal.pone.0235925)
Supplement: S1 Raw images — (PDF) [file pone.0235925.s001.pdf]

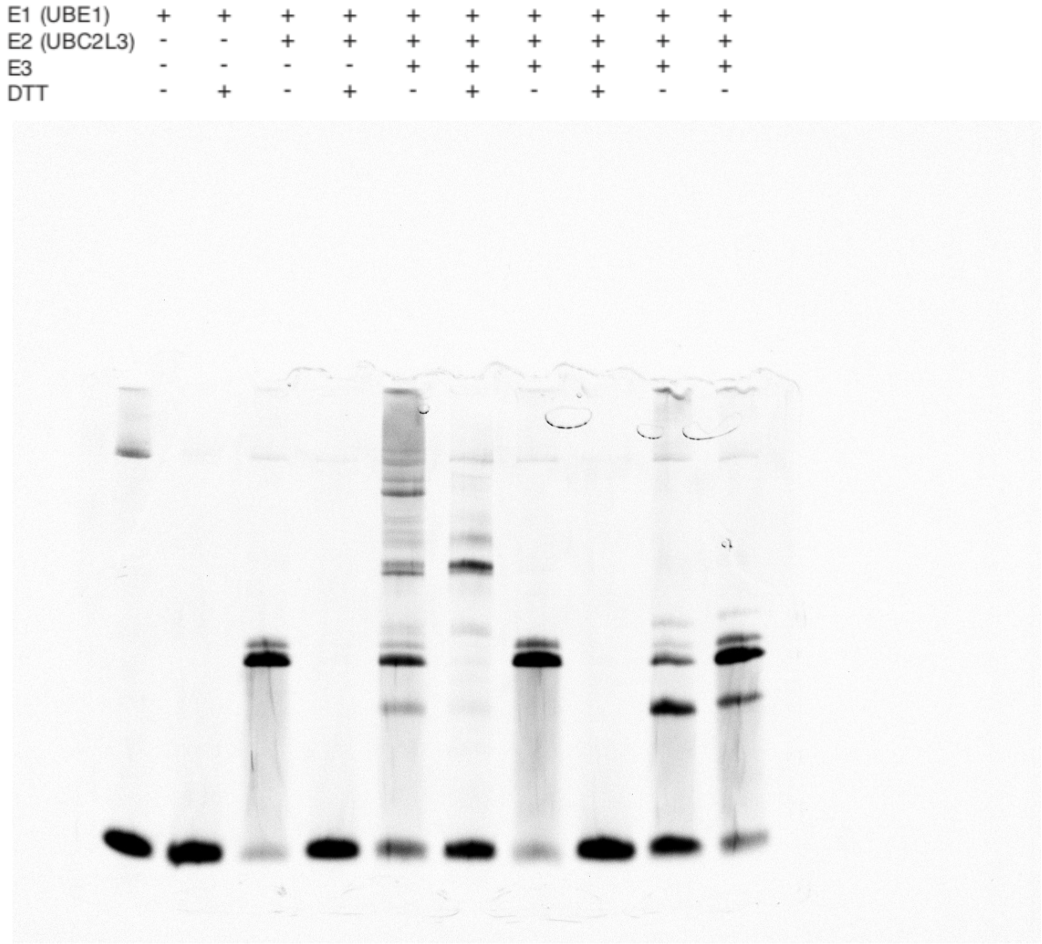


Original Image taken on iBright FL1000 gel image for AlexaFluor 647 (inverted to B+W).
16.5% Bis-Tris acrylamide gel
Each visible band N-terminally labeled AlexaFluor 647 ubiquitin alone (bottom band) or in covalent complex with E1, E2, or E6AP protein.

Components in each lane are as indicated above lanes and described in the text.

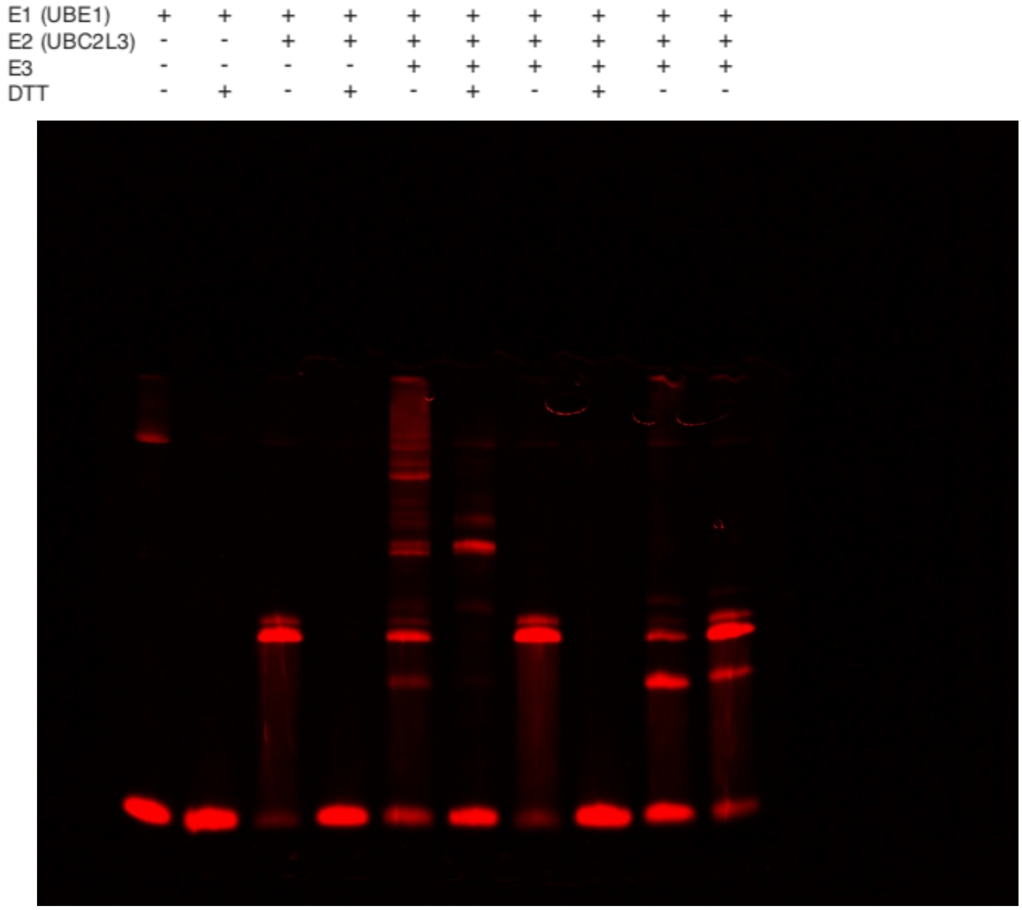


Original Image taken on iBright FL1000 gel image for AlexaFluor 647.
16.5% Bis-Tris acrylamide gel
Each visible band N-terminally labeled AlexaFluor 647 ubiquitin alone (bottom band) or in covalent complex with E1, E2, or E6AP protein.

Components in each lane are as indicated above lanes and described in the text.
